# Frailty Rather Than Sonographic Muscle Measurement Predicts Outcome in Liver Cirrhosis

**DOI:** 10.1002/jcsm.70373

**Published:** 2026-10-27

**Authors:** Wolfgang Maximilian Kremer, Alexander Schwarz, Marie‐Claire Dumont, Simon Johannes Gairing, Visvakanth Sivanathan, Peter Robert Galle, Martin Franz Sprinzl, Eva Maria Schleicher, Christian Labenz

**Affiliations:** ^1^ Department of Internal Medicine I University Medical Center of the Johannes Gutenberg‐University Mainz Germany

**Keywords:** clinical index frailty, frailty, muscle, sarcopenia, sonography, ultrasound

## Abstract

**Background:**

Sarcopenia and frailty are frequent in patients with liver cirrhosis and are associated with morbidity and mortality. Given the limitations of computed tomography (CT) for routine assessment of muscle loss and the need for practical bedside assessment, we aimed to compare sonographic muscle thickness measurement and the Clinical Frailty Scale (CFS) for their prognostic value in patients with liver cirrhosis.

**Methods:**

In this study, data of prospectively recruited patients with liver cirrhosis (both in‐ and outpatients) treated at a tertiary care centre were retrospectively analysed. Sonographic muscle thickness measurement was used to calculate the feather thigh muscle thickness index (fTMTI) and the compression thigh muscle thickness index (cTMTI). Frailty was assessed using the Clinical Frailty Scale. Patients were followed for liver transplantation or death.

**Results:**

In total, 153 patients were included with a median Model for End‐Stage Liver Disease (MELD) of 18 (Interquartile range (IQR) 10; 24). Ninety‐five patients (62.0%) were male, and 70 patients (46%) were recruited in the outpatient setting. During the 90‐day follow‐up period, 71 patients (46.4%) either died or underwent liver transplantation. Outpatients had more muscle mass than inpatients and a lower CFS (fTMTI 19.1 mm/m vs. 12.1 mm/m, *p* < 0.001 and CFS 3 vs. 5, *p* < 0.001). In multivariable Cox‐regression models adjusted for age and MELD score, a higher CFS remained independently associated with mortality and liver transplantation (LT) (HR 1.25 (1.14; 1.63), *p* < 0.001), whereas fTMTI and cTMTI were not consistently associated with a poorer prognosis (fTMTI HR 1.52 (0.68; 3.42), *p* = 0.300; cTMTI HR 1.01 (0.61; 1.68), *p* > 0.9). Subgroup analyses in men confirmed a superior prognostic value of CFS (HR 1.51 (1.17; 1.94), *p* = 0.001) over sonographic muscle thickness measurement (cTMTI HR 0.94 (0.84; 1.04), *p* = 0.200; fTMTI HR 0.93 (0.88; 1.00), *p* = 0.039).

**Conclusion:**

CFS is robustly associated with poorer prognosis in patients with liver cirrhosis and seems to be superior to sonographic muscle thickness measurement of the thigh.

**Abbreviations**
ALD: alcoholic liver disease
AUC: area under the curve
BMI: body mass index
CFS: clinical frailty scale
cm: centimetre
CT: computed tomography
cTMTI: compression thigh muscle thickness index
fTMTI: feather thigh muscle thickness index
HCC: hepatocellular carcinoma
INR: international normalized ratio
IQR: interquartile range (Q1; Q3)
kg: kilogram
MASLD: metabolic dysfunction‐associated steatotic liver disease
MELD: model for end‐stage liver disease
MRI: magnetic resonance imaging
n: number
SMI: skeletal muscle index
TMT: thigh muscle thickness
TMTI: thigh muscle thickness index
VC: variation coefficient
Wbc: white blood cells
yr: years

## 1 Introduction

Sarcopenia and frailty are increasingly recognized complications of liver cirrhosis and have been independently associated with adverse outcomes both before and after liver transplantation [1, 2, 3]. Although the Model for End‐Stage Liver Disease (MELD) score is still considered the gold standard for predicting mortality in patients with end‐stage liver disease, it does not capture the extrahepatic features of cirrhosis, such as muscle wasting, malnutrition and functional decline [4].

Frailty is an indicator of increased vulnerability to stressors, both physical and mental. It reflects a patient's ability to perform basic and instrumental activities of daily living and represents a clinical syndrome characterized by a decline in physical and physiological reserves, ultimately leading to vulnerability and adverse health outcomes [5, 6, 7]. Historically considered a condition of aging, frailty is now recognized across all chronic diseases, including liver cirrhosis. In patients with liver cirrhosis, frailty is associated with increased complications such as rehospitalization and increased mortality [8, 9].

According to the latest European Working Group on Sarcopenia in older People 2 guidelines (EWGSOP2), sarcopenia encompasses the loss of both muscle quantity and quality. In contrast to sarcopenia frailty captures a broader functional profile, integrating physical, cognitive and physiological dimensions [5, 6, 7]. Muscle strength is typically assessed by hand grip strength, whereas imaging modalities such as computed tomography (CT) or ultrasound allow quantification of muscle mass [10]. CT‐based measurements, such as the skeletal muscle index (SMI), are currently the gold standard for identifying sarcopenia and have been associated with hepatic encephalopathy, infections, ascitic decompensation and prognosis after transjugular intrahepatic portosystemic shunt (TIPS) placement [3, 11, 12, 13, 14, 15, 16, 17]. However, a CT scan is not always available to all patients and is not practical for routine bedside assessment in all patients. In this context, sonographic muscle thickness measurement has emerged as a promising, noninvasive, radiation‐free alternative. Sonographic assessment of the thigh is acknowledged as a reliable and accurate technique for measuring muscle size [18, 19]. It has already been used in the context of various diseases, and low muscle indices seem to be associated with poorer clinical outcomes [20, 21]. However, data on the predictive value of sonographic assessment of muscle mass in liver cirrhosis in relation to the CFS are currently limited and no direct comparisons are available [22].

Therefore, it was the aim of this study to compare the usefulness of both testing strategies, sonographic assessment of muscle mass and the CFS, in patients with liver cirrhosis and to evaluate their usefulness for predicting transplantation‐free survival.

## 2 Material and Methods

### 2.1 Patient Cohort

For this study, data from two prospectively enrolled cohorts were merged and retrospectively analysed. The first cohort included patients from the prospective ongoing LIVKID study. LIVDKID is a prospective observational study including hospitalized patients with decompensated cirrhosis and AKI treated at normal wards of the Cirrhosis Center of the University Medical Center Mainz, Germany. The patients included in this current study were recruited in LIVKID between April 2022 and June 2024.

The second cohort consisted of outpatients and inpatients with liver cirrhosis who underwent abdominal ultrasound in the ultrasound department of the Department of Internal Medicine I of the University Medical Center Mainz between March 2020 and June 2022.

The leading aetiology of the underlying liver disease was determined according to clinical, serological and histological findings. Diagnosis of cirrhosis was established by histology or a combination of conclusive appearance in ultrasound, radiological imaging, endoscopic features of portal hypertension and medical history. Blood biochemistry was assessed in all patients.

Patients with degenerative muscle diseases and those unable to provide informed consent were excluded from this study.

### 2.2 Sonographic Muscle Thickness Measurement

Muscle ultrasound was performed in all patients and was conducted using either the Aplio i800 ultrasound system (Canon, Tokyo, Japan) or the ACUSON Freestyle ultrasound system (Siemens Healthcare, Erlangen, Germany). The ultrasound measurements were performed in real time. The examiners were trained and instructed in advance for the ultrasound measurements. All ultrasound examinations were performed only by these examiners. Each measurement was displayed in an optimized B‐mode image.

All patients lay in supine position for sonographic examination, as body position affects muscle dimensions. The extensor muscle thickness of the lower limb, including the rectus femoris muscle and the vastus intermedius muscle, was measured in the frontal plane at the midpoint between the spina iliaca anterior and the cranial edge of the patella. The combined extensor muscle thickness was measured between the femur bone and the subcutaneous fat tissue, excluding the subcutaneous fat layer itself. Measurements were performed for both sides and the mean value of all measurements was calculated.

The thigh muscle thickness index (TMTI) represents the average bilateral thickness of the lower limb extensor muscles (rectus femoris muscle and vastus intermedius muscle), indexed to the patient's body height (mm/m). Measurements of the same thigh muscle group under compression, using an ultrasound detector, were used to calculate the compressed TMTI (cTMTI). The feather thigh muscle thickness index (fTMTI) represents the same measurement but without pressure using the ultrasound detector. The technique is also described elsewhere [23, 24, 25, 26]. Previously published gender‐specific reference threshold values were used to classify patients into low or high muscle mass categories. The cut‐offs were as follows: men: fTMTI 21.5 mm/m, cTMTI 6.0 mm/m and women: fTMTI 16.5 mm/m, cTMTI 8.1 mm/m [19].

### 2.3 Assessment of the Clinical Frailty Scale

For the CFS, the patient was asked about his ability to manage daily life during the 2 weeks prior to the examination. CFS has been used in various studies in patients with liver cirrhosis and is also described elsewhere [9, 27, 28]. The CFS is based on a clinical assessment and is divided into nine categories reflecting the patient's daily functioning: (1, *very fit*; 2, *well*; 3, *well with treated comorbid diseases*; 4, *apparently vulnerable*; 5, *mildly frail*; 6, *moderately frail*; 7, *severely frail*; 8, *very severely frail*; and 9, *terminally ill*). Each category is accompanied by explanatory images. Frailty is defined as a CFS score of 5 or higher (CFS 5–9), whereas at least prefrailty is defined as a CFS score of 4 or higher (CFS 4–9).

### 2.4 Follow‐Up

All patients were followed up for at least 90 days after study inclusion. The composite end point of death or liver transplantation was defined as the primary end point during the follow‐up. We decided to use this composite end point given that all patients who had received a liver transplantation had done so because of final hepatic failure.

### 2.5 Ethics

Written informed consent was obtained from the participants before inclusion in the study. The study was conducted in accordance with the ethical guidelines outlined in the 1975 Declaration of Helsinki and its subsequent amendments. Both study protocols were approved by the ethics committee of the Landesärztekammer Rheinland‐Pfalz (accession numbers 2022‐16356 and 2020‐15174).

### 2.6 Statistical Analyses

Statistical analyses and graphic design were performed with IBM SPSS Statistics Version 29.0 (Armonk, NY: IBM Corp.), R 4.1.3 (R Core Team, 2022) and GraphPad Prism Version 8.0.2 (GraphPad Software, California, USA).

Categorical variables are presented as numbers and percentages. Continuous variables are presented as medians (IQR). Normal distribution was tested using the Shapiro–Wilk normality test, and subsequent comparisons between groups were made using a Mann–Whitney *U* test, an unpaired *t*‐test or a chi‐squared test.

For liver transplantation‐free‐survival analyses, Kaplan–Meier curves and Cox proportional hazards regression models (univariable and multivariable) were constructed. The log‐rank test was used to test for differences between groups in Kaplan–Meier curve analysis. The cut‐off values for low and high muscle mass were taken from our previously published study [19]. Accordingly, they were included in both the univariate and multivariate analyses as dichotomous variables. Multivariable Cox‐regression models were adjusted for MELD, age and the respective muscle or frailty measurement. We did not include other variables to avoid overfitting.

To investigate how well cTMTI and fTMTI identify patients reaching the composite end point of death or LT within 90 days, we calculated the area under the receiver operating characteristic curve (AUROC) and its respective 95% confidence interval (CI). For cTMTI and fTMTI, we also determined the cut‐off value maximizing Youden's Index.

To evaluate the robustness of the primary findings, cumulative incidences of mortality under consideration of liver transplantation as a competing event were plotted for cTMTI and fTMTI. Additionally, a competing‐risk analysis was performed using fine and grey subdistribution hazard models with death as the outcome of interest and liver transplantation as a competing event. Multivariable models were adjusted for age and MELD score and included either the CFS or the respective sonographic muscle measurement. Results are reported as subdistribution hazard ratios (sHRs) with 95% confidence intervals.

For all tests, we used a 0.05 level to define statistically relevant deviations from the respective null hypotheses. Given that our complete data analysis is exploratory, *p*‐values must be interpreted in the context of the study design. No adjustments for multiple testing were performed.

## 3 Results

### 3.1 Study Cohort

A total of 153 patients with liver cirrhosis were analysed in this study. More than half of the cohort were male (62%) and the median age was 59 years. Of the 153 patients, 70 were recruited in the outpatient setting. The most common aetiology of liver cirrhosis was alcohol‐related (49.0%), followed by viral hepatitis (16.0%) and metabolic dysfunction‐associated steatotic liver disease (MASLD; 13.0%). The median MELD score was 18 (IQR: 10; 24). Sonographic muscle thickness measurement analysis revealed a median fTMTI of 14.8 mm/m (IQR: 10.9; 19.8) and a cTMTI of 8.3 mm/m (IQR: 5.8; 10.7). The median Clinical Frailty Scale (CFS) score was 3 (IQR: 2; 4). Additional baseline characteristics of the cohort are summarized in Table 1.

**TABLE 1:** Demographics and clinical characteristics of the cohort at the time of study inclusion.

Variable	Total cohort
*n*	153
Male, *n* (%)	95 (62.0)
Age, years (IQR)	59 (54; 64)
BMI, kg/m^2^ (IQR)	27.0 (23.9; 31.1)
Outpatients, *n* (%)	70 (46)
MELD, points (IQR)	18 (10; 24)
Child‐Pugh category (A/B/C), *n* (%)	54/54/45 (35/35/29)
Death or liver transplantation during follow‐up, *n* (%)	71 (46.4)
Aetiology	
Alcohol‐associated, *n* (%)	75 (49.0)
Viral hepatitis, *n* (%)	25 (16.0)
MASLD, *n* (%)	20 (13.0)
Other/mixed, *n* (%)	33 (22.0)
Sonographic muscle thickness measurement	
fTMTI, mm/m (no compression) (IQR)	14.8 (10.9; 19.8)
cTMTI, mm/m (compression) (IQR)	8.3 (5.8; 10.7)
Frailty assessment	
CFS (IQR)	3 (2; 4)
Laboratory tests	
Sodium, mmol/L (IQR)	137 (132; 140)
Creatinin, mg/dL (IQR)	1.4 (0.8; 2.1)
Bilirubin, mg/dL (IQR)	1.6 (0.9; 3.6)
Albumin, g/dL (IQR)	31 (25; 39)
INR, (IQR)	1.4 (1.2; 1.7)
WBC, /μL (IQR)	6.0 (4.6; 8.3)
Haemoglobin, g/dL (IQR)	11.0 (8.8; 13.5)
Platelets, /μL (IQR)	109 (71; 152)

*Note:* Data are expressed as medians and interquartile ranges or as frequencies and percentages.

Abbreviations: CFS, clinical frailty scale; cTMTI, compression thigh muscle thickness index; fTMTI, feather thigh muscle thickness index; INR, international normalized ratio; IQR, interquartile range; MASLD, metabolic dysfunction‐associated steatotic liver disease; MELD, model for end‐stage liver disease; WBC, white blood cells.

A comparison between inpatients and outpatients is displayed in Table S1. As expected, inpatients were in a more advanced stage of liver disease, as reflected by a significantly higher MELD score (median: 23 vs. 9; *p* < 0.001). Sonographic muscle thickness indices were significantly lower in the inpatient cohort: median fTMTI was 12.1 mm/m (9.2; 14.8) and cTMTI was 6.2 mm/m (5.2; 9.0), compared to 19.1 mm/m (15.4; 23.7; *p* < 0.001) and 10.6 mm/m (7.9; 12.3; *p* < 0.001) in outpatients, respectively. In line, CFS scores were higher in inpatients than in outpatients (median score 5 vs. 3; *p* < 0.001).

Comparisons between patients with low or high muscle mass according to the established cut‐offs for fTMTI and cTMTI are displayed in Tables S2 and S3. Patients in the high muscle mass group according to fTMTI were significantly younger (53 vs. 60 years; *p* < 0.0001) and had a higher BMI (29.3 vs. 26.3 kg/m^2^; *p* < 0.001).

### 3.2 Identification of Ideal Cut‐Offs of fTMTI and cTMTI for Predicting the Composite End Point of Death/Liver Transplantation (LT)

To analyse the discriminate ability of fTMTI and cTMTI for predicting the composite end point of death/LT within 90 days, we determined the AUC of ROC curves. Of note, both fTMTI and cTMTI were analysed separately in women and men due to differences in muscle mass between sexes. Overall, AUCs were modest. The highest discriminative performance was observed for fTMTI in women, with an AUC of 0.73 (Figure 1). The lowest value was seen for cTMTI in men, with an AUC of 0.67. The corresponding ROC curves are presented in Figure 1. The ideal cut‐offs for predicting death/LT were derived according to the Youden's index. The respective cut‐offs were as follows: female fTMTI: 15.20 mm/m, female cTMTI: 6.61 mm/m, male fTMTI 14.02 mm/m and male cTMTI: 9.55 mm/m.

**FIGURE 1:**
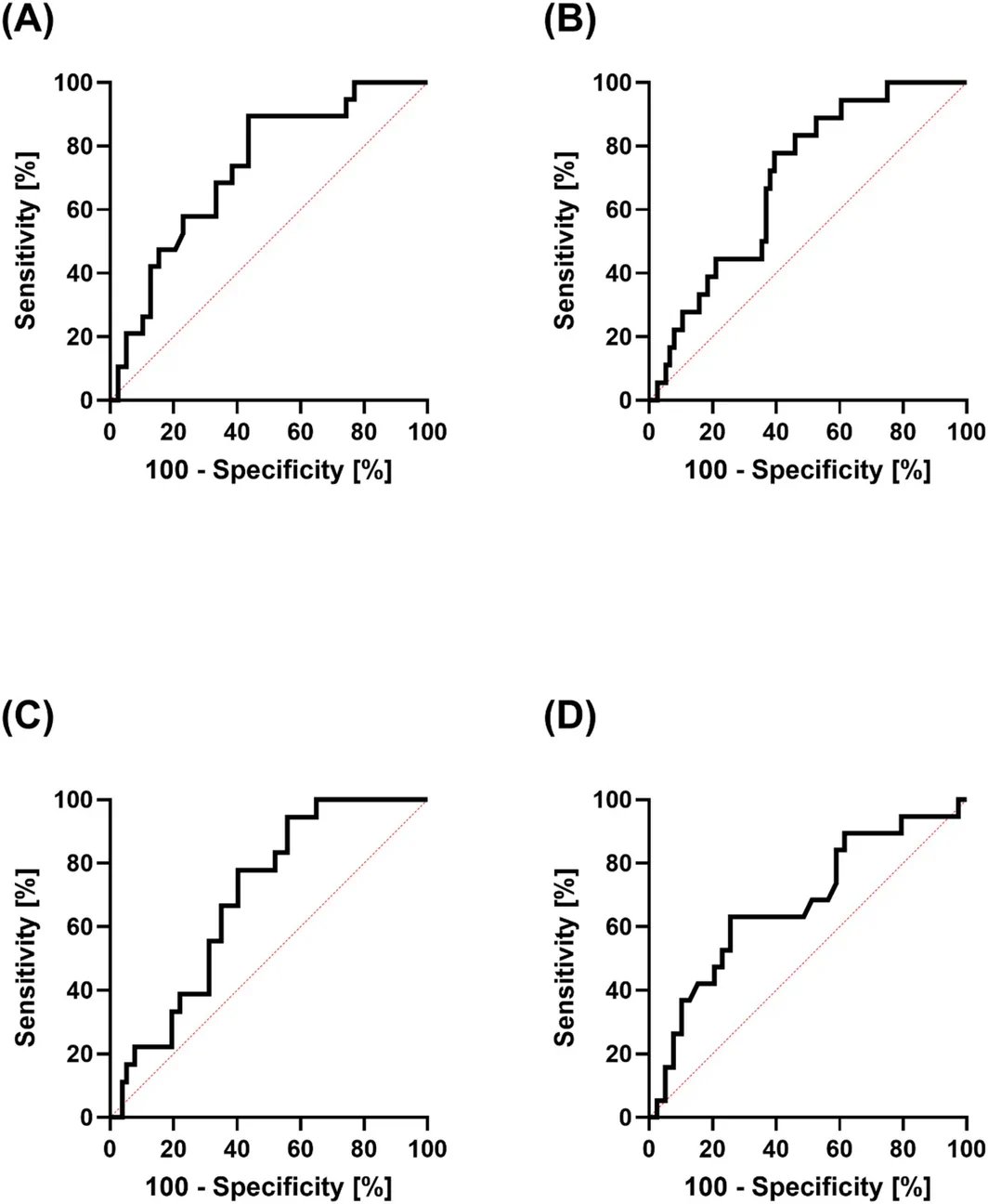
Receiver operating characteristic (ROC) curve of fTMTI and cTMTI for prediction of the composite end point of death/LT within 90 days. Figure 1A displays the ROC curve of fTMTI in females (AUC: 0.73). Figure 1B displays the ROC curve of fTMTI in males (AUC: 0.70). Figure 1C displays the ROC curve of cTMTI in females (AUC: 0.69). Figure 1D displays the ROC curve of cTMTI in males (AUC: 0.67).

### 3.3 Prediction of LT‐Free Survival

Median follow‐up was 90 days. During the follow‐up period 46.4% of the patients reached the composite end point of death (*n* = 41) or liver transplantation (*n* = 30). First, we conducted Kaplan–Meier plots to investigate the univariable impact of fTMTI or cTMTI dichotomized by established cut‐offs on LT‐free survival. Here, low muscle mass defined by fTMTI and cTMTI was associated with poorer LT‐free survival (Figure 2). This finding was also true in univariable Cox‐regression analyses (Table 2). CFS was also associated with poorer prognosis in univariable Cox‐regression analysis (Table 2).

**FIGURE 2:**
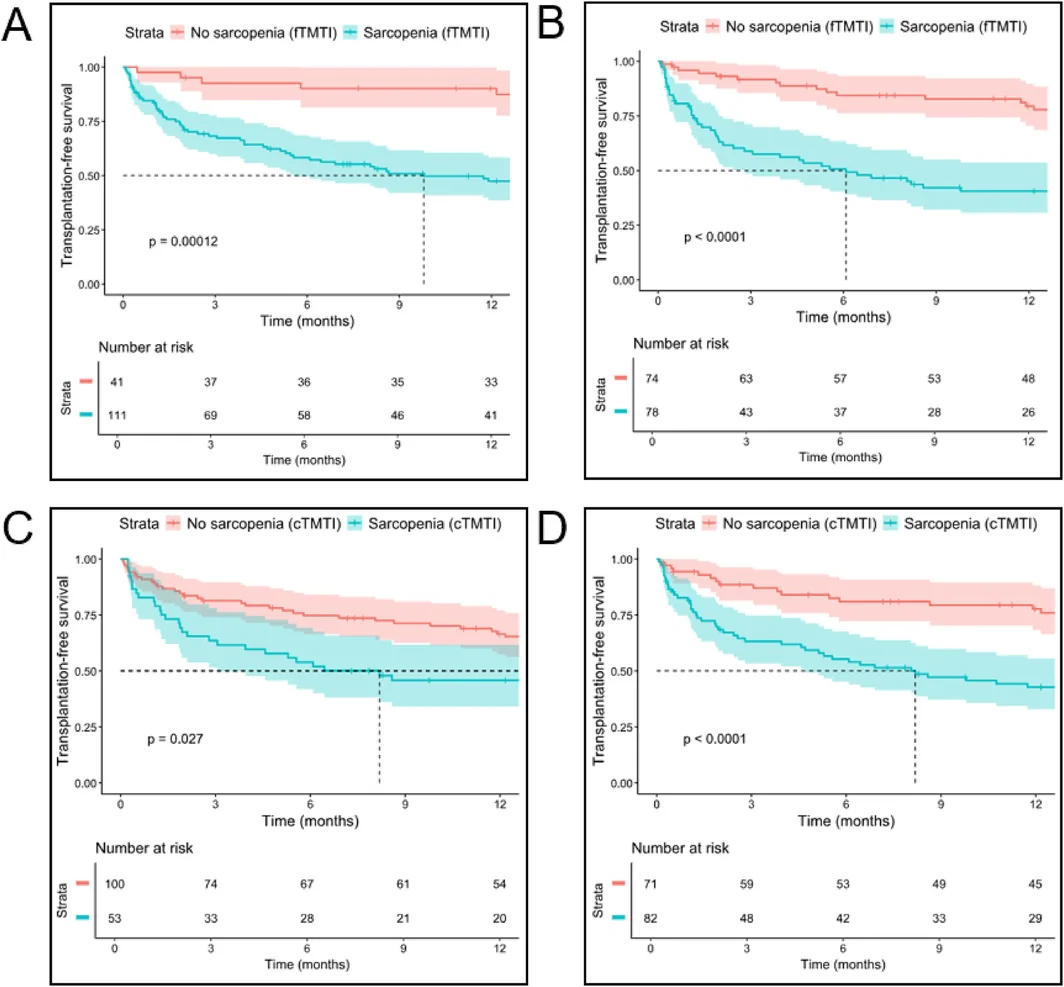
Liver transplantation‐free survival of all patients stratified by muscle indices in sarcopenia and no sarcopenia. Kaplan Meier Curves with liver transplantation‐free survival. Seventy‐one patients (46.4%) either died or underwent liver transplantation. (A) stratification according to established cut‐off values for fTMTI (HR 3.46 (95% CI 1.76; 6.78), *p* < 0.001). (B) Stratification according to optimized cut‐off values for fTMTI (HR 3.40 (95% CI 2.03; 5.69), *p* < 0.001); (C) stratification according established cTMTI (HR 1.70 (95% CI 1.06; 2.72), *p* = 0.028), (D) Stratification according to optimized cut‐off values for cTMTI (HR 2.73 (95% CI 1.65; 4.53), *p* < 0.001).

**TABLE 2:** Univariable Cox‐regression analyses for variables associated with death or liver transplantation in the total cohort.

Variable	Univariable analysis	
	**Hazard ratio (95%‐CI)**	*p*
Age, *y*	1.00 (0.98; 1.03)	0.7
Gender, male	0.82 (0.51; 1.31)	0.4
Alcohol cirrhosis (vs. other aetiologies)	0.5 (0.15; 1.70)	0.3
Child‐Pugh‐Score B	6.2 (2.73; 13.3)	**< 0.001**
MELD score, points	1.13 (1.10; 1.16)	**< 0.001**
Sodium, mmol/L	0.87 (0.84; 0.90)	**< 0.001**
Creatinine, mg/dL	1.84 (1.56; 2.18)	**< 0.001**
Bilirubin, mg/dL	1.05 (1.02; 1.08)	**< 0.001**
Albumin, g/L	0.91 (0.88; 0.94)	**< 0.001**
WBC, /μL	1.02 (1.00; 1.04)	0.094
Haemoglobin, g/dL	0.69 (0.63; 0.76)	**< 0.001**
Platelets, /μL	0.99 (0.99; 1.0)	**0.005**
INR, (INR)	2.14 (1.52; 3.02)	**< 0.001**
Established fTMTI, mm/m	3.46 (1.76; 6.78)	**< 0.001**
Established cTMTI, mm/m	1.70 (1.06; 2.72)	**0.028**
Optimized fTMTI, mm/m	3.40 (2.03; 5.69)	**< 0.001**
Optimized cTMTI, mm/m	2.73 (1.65; 4.53)	**< 0.001**
CFS	1.53 (1.32; 1.78)	**< 0.001**

*Note: p*‐values in bold designate statistical significance of < 0.05.

Abbreviations: CFS, clinical frailty scale; cTMTI, compression thigh muscle thickness index; fTMTI, feather thigh muscle thickness index; INR, international normalized ratio; MELD, model for end‐stage liver disease; WBC, white blood cell count.

To identify variables independently associated with a poor LT‐free survival, we conducted multivariable Cox‐regression analyses. Here, low muscle mass defined by established threshold values for cTMTI and fTMTI was not independently associated with poorer prognosis after adjusting for MELD and age (Table 3). In contrast, CFS (HR 1.36, 95% CI 1.14; 1.63, *p* < 0.001) and low muscle mass defined according to the newly derived fTMTI‐cut‐off (HR 1.82, 95% CI 1.00; 3.30; *p* = 0.049) were independently associated with poorer prognosis after adjusting for MELD and age in the total cohort (Table 3).

**TABLE 3:** Multivariable Cox‐regression analyses for variables associated with death or liver transplantation in the total cohort.

Variable	Hazard ratio (95% CI)	*p*
**Total cohort**		
**Model 1**		
Age, years	1.00 (0.98; 1.03)	0.7
Meld score	1.11 (1.08; 1.15)	**< 0.001**
CFS	1.36 (1.14; 1.63)	**< 0.001**
Model 2*		
fTMTI dichotomized, mm/m	1.52 (0.68; 3.42)	0.300
Model 3*		
cTMTI dichotomized, mm/m	1.01 (0.61; 1.68)	> 0.9
Model 4*		
Optimized fTMTI, mm/m	1.82 (1.00; 3.30)	**0.049**
Model 5*		
Optimized cTMTI, mm/m	1.36 (0.77; 2.38)	0.3

*Note: p*‐values in bold designate statistical significance of < 0.05. Established fTMTI and cTMTI refer to the thresholds for muscle indices calculated from a previous cohort using the SMI [19]. Optimized thresholds specific to liver cirrhosis were derived for the cohort used in this study.

Abbreviations: CFS, clinical frailty scale; cTMTI, compression thigh muscle thickness index; fTMTI, feather thigh muscle thickness index; MELD, model for end‐stage liver disease.

* all models were adjusted for MELD score and age.

To further investigate the usefulness of fTMTI, cTMTI and CFS in sicker patients, we conducted subgroup analyses in inpatients. Here, low muscle mass defined by the established or the optimized cut‐offs was not associated with poorer prognosis in multivariable analyses (Table 4). In contrast, a higher CFS was significantly and independently associated with LT‐free survival after adjusting for MELD and age in men (HR 1.51; CI 1.17; 1.94, *p* = 0.001) (Table 4). Subgroup analyses in the outpatient cohort were omitted due to the lack of events.

**TABLE 4:** Multivariable Cox‐regression analyses for variables associated with death or liver transplantation in females, males and inpatients.

Variable	Hazard ratio (95% CI)	*p*
**Males**		
**Model 1**		
Age, years	1.00 (0.97; 1.03)	0.9
MELD score, points	1.11 (1.06; 1.16)	**< 0.001**
CFS, points	1.51 (1.17; 1.94)	**0.001**
Model 2*		
Established cTMTI, mm/m	0.94 (0.84; 1.04)	0.2
Model 3*		
Established fTMTI, mm/m	0.93 (0.88; 1.00)	**0.039**
**Females**		
**Model 1**		
Age, years	1.02 (0.098; 1.06)	0.3
MELD score, points	1.13 (1.08; 1.18)	**< 0.001**
CFS, points	1.13 (0.7; 1.48)	0.3
Model 2*		
Established cTMTI, mm/m	1.23 (1.06; 1.43)	**0.007**
Model 3*		
Established fTMTI, mm/m	1.10 (1.00; 1.21)	0.051
**Inpatients**		
**Model 1**		
Age, years	1.02 (0.99; 1.04)	0.3
MELD score, points	1.06 (1.02; 1.11)	**0.005**
CFS, points	1.24 (0.099; 1.54)	0.058
Model 2*		
Established cTMTI, mm/m	1.02 (0.95; 1.10)	0.6
Model 3*		
Established fTMTI, mm/m	1.00 (0.05; 1.05)	0.9
Model 4*		
Optimized cTMTI, mm/m	1.12 (0.60; 2.09)	0.7
Model 5*		
Optimized fTMTI, mm/m	1.14 (0.56; 2.29)	0.7

*Note: p*‐values in bold designate statistical significance of < 0.05. Established fTMTI and cTMTI refer to the thresholds for muscle indices calculated from a previous cohort using the SMI [19]. Optimized thresholds specific to liver cirrhosis were derived for the cohort used in this study.

Abbreviations: CFS, clinical frailty scale; cTMTI, compression thigh muscle thickness index; fTMTI, feather thigh muscle thickness index; MELD, model for end‐stage liver disease.

* all models were adjusted for MELD score and age.

### 3.4 Prediction of Survival

As a sensitivity analysis, mortality was evaluated as the end point, whereas liver transplantation was considered a competing event. Cumulative incidence plots for fTMTI and cTMTI are displayed in Figure S1. In multivariable analyses adjusted for age and MELD score, the CFS remained independently associated with mortality (HR 1.61, 95% CI 1.29–2.00, *p* < 0.001). In contrast, neither established nor optimized cTMTI and fTMTI thresholds were associated with mortality in any model (all *p* ≥ 0.25) (Table S4).

## 4 Discussion

Risk stratification in patients with cirrhosis is important to improve organ allocation strategies and therapeutic interventions. In this study, the CFS emerged as a robust predictor of poorer prognosis in patients with liver cirrhosis and seems to be superior to ultrasound muscle thickness measurement of the thigh muscle.

Muscle wasting is a well‐described complication of advanced liver disease and is associated with increased mortality and posttransplant morbidity [3, 11, 12]. In our study, reduced muscle mass of the thigh was associated with poorer outcomes in univariable analyses. However, most of the indices did not turn out to be associated with poorer prognosis in multivariable analyses after adjusting for MELD and age. This was especially true when using previously established cut‐offs derived from patients without cirrhosis. However, using optimized cut‐offs for this specific cohort, fTMTI turned out to be associated with poorer prognosis. The consistency of the results across both transplant‐free survival and competing‐risk mortality analyses underscores the superiority of frailty assessment over sonographic muscle measurements for risk stratification in advanced liver cirrhosis. Though, it has to be acknowledged that no validation cohort was available for this current study and therefore, these cut‐offs and the respective results have to be interpreted with caution. All in all, the CFS remained a more robust and consistent predictor across all analyses.

These findings raise important considerations regarding the utility of sonographic muscle thickness measurement of the thigh for detecting complications in patients with liver cirrhosis. Low muscle thickness of the leg may especially reflect systemic decompensation rather than serving as an independent driver of prognosis. Although sonographic sarcopenia assessment of the thigh may be useful as part of a broader frailty or nutritional evaluation, it might not add additional benefit in isolation to guide clinical decisions. The CFS, on the other hand, which is also easily applicable—even in bedside settings—and seems to be more consistent for identification of patients with poorer prognosis. The CFS integrates multiple domains of patient vulnerability, including physical function, activity level, cognition and physical reserve [29, 30]. In the past, it has been demonstrated that CFS is a reliable predictor of mortality and LT‐free survival in various studies, although its usefulness in compensated outpatients for prediction of decompensation or mortality is less well defined [9, 31].

It has to be acknowledged that our study results cannot be extrapolated to every definition of sarcopenia. Most published studies used the SMI, which usually relies on CT scans and has been linked to poorer prognosis in various settings [14, 32, 33, 34]. Given that CT scans were not readily available in all our patients, we cannot provide SMI results for this cohort and are therefore unable to compare the sonographic muscle measurements of the thigh with SMI in our cohort. The difference in prognostic utility between cTMTI/fTMTI and SMI may be explained by the fact that SMI measures trunk muscles, which may be more relevant for overall physical function than isolated assessment of the quadriceps muscle.

Sarcopenia defined by ultrasound indices has been investigated in liver cirrhosis in previous, smaller studies. Gödiker et al. examined a group of 62 patients with liver cirrhosis measuring the thigh muscle index [35]. Sarcopenic patients more frequently reached the primary end point of hepatic decompensation and died more frequently within 1 year. Notable differences between the study by Gödiker et al. and our current one are differences in the frequency of outcomes. In the aforementioned study, only nine patients died, limiting these analyses, whereas in our study 71 patients reached the composite end point of death or LT. Additionally, we used gender‐specific thresholds accounting for sex differences. Another recent study also investigated the influence of ultrasound measurement of the rectus femoris cross‐sectional area on complications such as hepatic decompensation or 30‐ and 90‐day mortality in 203 patients [22]. In this cohort, a lower rectus femoris cross‐sectional area was independently associated with poorer clinical outcomes. Given that we measured fTMTI and cTMTI in our study, both studies are not easily comparable. However, it might be possible that the rectus femoris cross‐sectional area might be a better parameter to predict prognosis in patients with cirrhosis. Future prospective studies should include various ultrasound muscle measurements to conduct proper comparisons.

An additional advantage of the CFS is that it can be assessed repeatedly during follow‐up, allowing longitudinal evaluation of a patient's functional status. Future studies should investigate whether improvements in frailty through targeted interventions such as rehabilitation, exercise training or nutritional support translate into improved clinical outcomes. If confirmed, frailty assessment may not only serve as a prognostic marker but also as a clinically meaningful target for intervention in patients with advanced cirrhosis.

This study has limitations that have to be acknowledged. First, the study was conducted at a single centre, which may limit the generalizability of our findings. Second, we used centre‐specific thresholds that have not yet been validated in large external cohorts. Thirdly the lack of validation in an independent cohort is noted. Although the prospective design and the consistency of the findings across multiple analyses support the robustness of our results, external validation in larger multicentre cohorts is warranted before broad generalization of these findings. Finally, we did not include complementary functional measures of sarcopenia or frailty, such as handgrip strength or the Liver Frailty Index, and therefore a direct comparison with these measures is not possible in the current study.

In conclusion, we found that the CFS is a robust predictor for poorer prognosis in patients with liver cirrhosis and seems to be superior to ultrasound muscle thickness measurement of the thigh muscle. Therefore, implementation of CFS in routine clinical practice of physicians treating patients with cirrhosis seems to be recommendable, whereas further studies are needed to elucidate the role of ultrasound definitions of sarcopenia.

## Funding

The authors have nothing to report.

## Ethics Statement

The study was conducted according to the ethical guidelines of the 1964 Declaration of Helsinki and its later amendments. The study protocols were approved by the ethics committee of the Landesärztekammer Rheinland‐Pfalz (accession number 2022‐16356 and 2020‐15174).

## Conflicts of Interest

The authors declare no conflicts of interest.

## Supporting information


**Table S1:** Comparison of outpatients and inpatients.
**Table S2:** Total cohort divided in muscle loss according to fTMTI.
**Table S3:** Total cohort divided in muscle loss according to cTMTI.
**Table S4:** Multivariable fine and grey competing‐risk regression analyses for mortality with liver transplantation as competing event in all patients.
**Figure S1:** Cumulative incidence of death stratified by muscle indices in sarcopenia and no sarcopenia.

## Data Availability

The data that support the findings of this study are available on request from the corresponding author. The data are not publicly available due to privacy or ethical restrictions.
