# Supplementary material for: Frailty Rather Than Sonographic Muscle Measurement Predicts Outcome in Liver Cirrhosis
Source: J Cachexia Sarcopenia Muscle. 2026 Oct 27;17(6):e70373. doi: 10.1002/jcsm.70373 (PMC13711254; doi:10.1002/jcsm.70373)
Supplement: Supplementary file 1 [file JCSM-17-e70373-s001.docx]

**Supplementary tables**

**Table S1.** **Comparison of outpatients and inpatients**

| **Variable** | **Outpatients** | **Inpatients** | **p-value** |
| --- | --- | --- | --- |
| **N** | **70** | **83** |  |
| male, n (%) | 42 (60.0) | 53 (63.9) | 0.373 |
| Age, years (IQR) | 59 (53; 65) | 59 (54; 64) | 0.046 |
| BMI, kg/m² (IQR) | 27.3 (24.0; 31.5) | 26.7 (23.3; 30.6) | 0.118 |
| MELD, points (IQR) | 9 (7; 14) | 23 (19; 31) | < 0.001 |
| Child-Pugh category (A/B/C), n (%) | 53/17/0 (75.7/17.1/0.0) | 1/37/45 (1.2/44.6/54.2) | < 0.001 |
| Death or liver transplantation, n (%) | 12 (17.1) | 59 (71.1) | < 0.001 |
| **Etiology** | | | |
| Alcohol, n (%) | 24 (34.3) | 51 (61.4) | 0.003 |
| Viral hepatitis, n (%) | 13 (18.6) | 12 (14.5) | 0.003 |
| MAFLD, n (%) | 15 15 (21.4) | 5 (6.0) | 0.003 |
| Other/mixed, n (%) | 18 (25.7) | 15 (18.0) | 0.003 |
| **Morphometric variables** | | | |
| fTMTI, mm/m (no compression) (IQR) | 19.1 (15.4; 23.7) | 12.1 (9.2; 14.8) | < 0.001 |
| cTMTI, mm/m (compression) (IQR) | 10.6 (7.9; 12.3) | 6.2 (5.3; 9.0) | < 0.001 |
| **Functional tests** | | | |
| CFS (IQR) | 3 (2; 4) | 5 (4; 6) | < 0.001 |
| **Laboratory** | | | |
| Sodium, mmol/L (IQR) | 140 (138; 141) | 133 (130; 137) | < 0.001 |
| Creatinine, mg/dl (IQR) | 0.8 (0.7; 0.9) | 2.0 (1.9; 2.8) | < 0.001 |
| Bilirubin, mg/dl (IQR) | 1.0 (0.8; 1.8) | 2.8 (1.2; 6.0) | < 0.001 |
| Albumin, g/dl (IQR) | 39 (32.8; 42.0) | 25.5 (22.0; 31.0) | < 0.001 |
| INR, (IQR) | 1.2 (1.1; 1.3) | 1.6 (1.4; 1.9) | < 0.001 |
| WBC, /µl (IQR) | 5.2 (4.1; 7.0) | 6.8 (5.1; 11.1) | < 0.001 |
| Hemoglobin, g/dl (IQR) | 14.0 (12.5; 15.6) | 9.0 (7.9; 10.5) | < 0.001 |
| Platelets, /µl (IQR) | 113 (74; 169) | 102 (62; 140) | < 0.001 |

BMI (body mass index), CFS (clinical frailty scale), cTMTI (compression thigh muscle thickness index), fTMTI (feather thigh muscle thickness index), INR (international normalized ratio), IQR (interquartile range), MASLD (metabolic dysfuntion-associated steatotic liver disease), MELD (model for end-stage liver disease), n (number), wbc (white blood cells), yr (years)

**Table S2. Total cohort divided in muscle loss according to fTMTI**,

| **Variable** | **Cohort** | | **p-value** |
| --- | --- | --- | --- |
|  | **High muscle mass according to fTMTI** | **Low muscle mass according to fTMTI** |  |
| Male gender, n (%) | 22 (54.0) | 72 (65) | 0.200 |
| Age, yr (IQR) | 53 (45; 60) | 60 (55;66) | **< 0.001** |
| BMI, kg/m² (IQR) | 29.3 (26.0; 34.3) | 26.3 (23.1; 30.1) | **< 0.001** |
| Setting, outpatient, n (%) | 36 (88) | 34 (31) | **< 0.001** |
| MELD, points (IQR) | 10 (7; 12) | 20 (14; 24) | **< 0.001** |
| Child Pugh A, B, C, n (%) | 29 (71.0), 9 (22.0), 3 (7.3) | 25 (23.0), 44 (40), 42 (38) | **< 0.001** |
| Etiology, ALD, MASLD, viral, n (%) | 13 (32), 9 (22), 10 (24) | 61 (55), 11 (9.9), 15 (14) | **0.030** |
| HCC, n (%) | 5 (12.0) | 16 (14.0) | 0.368 |
| History of ascites, n (%) | 11 (27.0) | 68 (61.0) | **< 0.001** |
| CFS, points (IQR) | 3 (2; 4) | 4 (3; 6) | **< 0.001** |
| Bilirubin, mg/dl (IQR) | 1.2 (0.9; 2.4) | 1.7 (0.9; 4.1) | 0.100 |
| Albumin, g/dl (IQR) | 38 (30; 41) | 29 (24; 36) | **< 0.001** |
| INR, (IQR) | 1.2 (1.1; 1.5) | 1.5 (1.3; 1.8) | **< 0.001** |
| Platelets, /µl (IQR) | 97 (71; 148) | 111 (71; 156) | 0.700 |

P-values in bold designate statistical significance of <0.05 BMI (body mass index), ALD (alcoholic liver disease), CFS (clinical frailty scale), fTMTI (feather thigh muscle thickness index), HCC (hepatiocellular carcinoma), INR (international normalized ratio), IQR (interquartile range), MASLD (metabolic dysfuntion associated steatotic liver disease), MELD (model for end-stage liver disease), n (number), wbc (white blood cells), yr (years)

**Table S3. Total cohort divided in muscle loss according to cTMTI**,

| **Variable** | **Cohort** | | **p-value** |
| --- | --- | --- | --- |
|  | **High muscle mass according to cTMTI** | **Low muscle mass according to cTMTI** |  |
| Male gender, n (%) | 71 (71.0) | 24 (45) | **0.002** |
| Age, yr (IQR) | 58 (52; 63) | 61 (57; 67) | **0.003** |
| BMI, kg/m² (IQR) | 28.4 (26.0; 32.1) | 23.4 (21.3; 27.9) | **< 0.001** |
| Setting, outpatient, n (%) | 58 (58.0) | 12 (23.0) | **< 0.001** |
| MELD, points (IQR) | 16 (8; 23) | 20 (15; 24) | **0.012** |
| Child Pugh A, B, C, n (%) | 46 (46.0), 30 (30.0), 24 (24.0) | 8 (15.0), 24 (45.0), 21 (40.0) | **< 0.001** |
| Etiology, ALD, MASLD, viral, n (%) | 49 (49.0), 17 (17.0), 17 (17.0) | 26 (49.0), 3 (5.7), 8 (15) | 0.100 |
| HCC, n (%) | 14 (14.0) | 18 (15.0) | 0.900 |
| History of ascites, n (%) | 45 (45.0) | 35 (66.0) | **0.013** |
| CFS, points (IQR) | 3 (2; 5) | 5 (3; 6) | **< 0.001** |
| Bilirubin, mg/dl (IQR) | 1.6 (0.9; 3.5) | 1.6 (0.8; 3.8) | 0.900 |
| Albumin, g/dl (IQR) | 32 (25; 40) | 31 (25; 36) | 0.400 |
| INR, (IQR) | 1.3 (1.1; 1.7) | 1.5 (1.2; 1.7) | 0.200 |
| Platelets, /µl (IQR) | 107 (74; 140) | 113 (67; 164) | 0.600 |

P-values in bold designate statistical significance of <0.05, ALD (alcoholic liver disease), BMI (body mass index), CFS (clinical frailty scale), cTMTI (compression thigh muscle thickness index), HCC (hepatiocellular carcinoma), INR (international normalized ratio), IQR (interquartile range), MASLD (metabolic dysfuntion associated steatotic liver disease), MELD (model for end-stage liver disease), n (number), wbc (white blood cells), yr (years)

**Table S4. Multivariable Fine and Gray competing-risk regression analyses for mortality with liver transplantation as competing event in all patients**

| **Variable** | **Subdistribution hazard ratio (95% CI)** | **p-value** |
| --- | --- | --- |
| **Males** | | |
| **Model 1** | | |
| Age, years | 1.02 (0.99; 1.06) | 0.2 |
| MELD-Score, points | 1.08 (1.04; 1.12) | **< 0.001** |
| CFS, points | 1.61 (1.29; 2.00) | **< 0.001** |
| **Model 2*** | | |
| Established cTMTI, mm/m | 1.02 (0.54; 1.93) | > 0.9 |
| **Model 3*** | | |
| Established fTMTI, mm/m | 1.00 (0.35; 2.86) | > 0.9 |
| **Model 4*** | | |
| Optimized cTMTI, mm/m | 1.50 (0.74; 3.01) | 0.3 |
| **Model 5*** | | |
| Optimized fTMTI, mm/m | 1.04 (0.51; 2.12) | > 0.9 |

P-values in bold designate statistical significance of <0.05

*all models were adjusted for MELD score and age

Established fTMTI and cTMTI refer to the thresholds for muscle indices calculated from a previous cohort using the SMI [19]. Optimized thresholds specific to liver cirrhosis were derived for the cohort used in this study. CFS (clinical frailty scale), cTMTI (compression thigh muscle thickness index), fTMTI (feather thigh muscle thickness index), MELD (model for end-stage liver disease)

**Figure S1. Cumulative incidence of death stratified by muscle indices in sarcopenia and no sarcopenia.**

Competing risk analysis with liver transplantation as competing event. S1A: stratification according to established cut-off values for fTMTI (p = 0.011). S1B: stratification according to optimized cut-off values for fTMTI (p = 0.010); S1C: stratification according established cTMTI (p = 0.13), S1D: stratification according to optimized cut-off values for cTMTI (p = 0.003)
